# Macromolecular Dyes by Chromophore-Initiated Ring Opening Polymerization of L-Lactide

**DOI:** 10.3390/polym12091979

**Published:** 2020-08-31

**Authors:** Francesca Cicogna, Guido Giachi, Luca Rosi, Elisa Passaglia, Serena Coiai, Roberto Spiniello, Federico Prescimone, Marco Frediani

**Affiliations:** 1 Consiglio Nazionale delle Ricerche Istituto di Chimica dei Composti OrganoMetallici, Sede Secondaria di Pisa (CNR ICCOM-SS-Pisa), 56124 Pisa, Italy; elisa.passaglia@pi.iccom.cnr.it (E.P.); serena.coiai@pi.iccom.cnr.it (S.C.); roberto.spiniello@pi.iccom.cnr.it (R.S.); 2 Dipartimento di Chimica “U. Schiff” Università degli Studi di Firenze, 50019 Sesto Fiorentino, Italy; guidogiachi@gmail.com (G.G.); luca.rosi@unifi.it (L.R.); marco.frediani@unifi.it (M.F.); 3 Dipartimento di Chimica e Chimica Industriale Università di Pisa, 56126 Pisa, Italy; federico.prescimone@bo.ismn.cnr.it; 4 Consiglio Nazionale delle Ricerche Istituto per lo Studio dei Materiali Nanostrutturati Sede Secondaria di Bologna, (CNR ISMN-SS-Bologna CNR), 40129 Bologna, Italy

**Keywords:** ring opening polymerization of L-lactide (RPO), chromophores end-capped PLA, dyed PLA, photophysical properties, migration tests, microwave-assisted polymerization

## Abstract

End functionalized polylactides are prepared by ring opening polymerization of L-lactide in the presence of stannous octoate (Sn(Oct)_2_). Three chromophores, 9H-carbazol-ethanol (CA), 9-fluorenyl-methanol (FM), and 2-(4-(2-chloro-4-nitrophenylazo)-N-ethylphenylamino)ethanol (Disperse Red 13, DR), are for the first time used as co-initiators in the polymerization process. The polymerization reaction is initiated by conventional thermal treatment, but in the case of FM, microwave-assisted polymerization is also carried out. CA and FM absorb and emit in the UV portion of the electromagnetic spectrum, whereas DR absorbs in the visible part. The obtained end-capped polylactides derivatives show the same photophysical properties as the initiator, so they are “macromolecular dyes” (MDs) that can be used “as synthesized” or can be blended with commercial poly(lactic acid) (PLA). The blends of PLA with MDs have ultraviolet-visible (UV-Vis) absorption and fluorescence emission features similar to that of MDs and thermal properties typical of PLA. Finally, migration tests, carried out onto the blends of PLA with MDs and PLA with free chromophores, show that MDs are less released than free chromophores both in solution and in the solid phase.

## 1. Introduction

**Figure 1:**
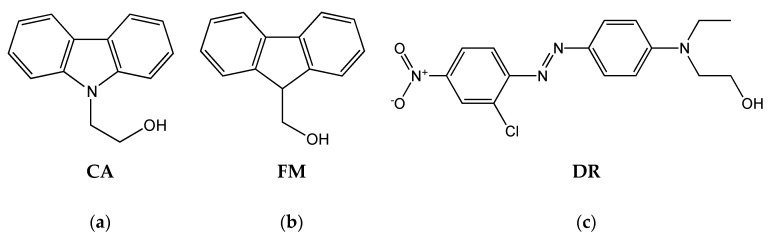
(**a**) 9H-carbazol-ethanol (CA), (**b**) 9-fluorenyl-methanol (FM), and (**c**) 2-(4-(2-chloro-4-nitrophenylazo)-N-ethylphenylamino)ethanol (Disperse Red 13, DR).

Within the last decade, rising environmental concerns are contributing to increasing commercial interest over renewable and biodegradable polymers. In this context, poly(lactic acid) (PLA) is gaining more and more importance, and therefore, a widening of its properties is nowadays highly desirable. While a lot of research is focusing on improving its physicochemical and mechanical properties, fewer efforts are made to enhance and modify PLA optical features in the UV-Vis region. PLA features make it particularly useful for the production of fibers, packaging, and other applications; therefore, the final UV-barrier capabilities and aesthetic appearance of polymers are topic issues having strong influence on the performances of products [1]. PLA films absorb in the lower range of the electromagnetic spectrum but are almost transparent to radiations in the UV-A and UV-B regions (85% of light is transmitted at wavelength (λ) about 250 nm; 95% of light is transmitted at λ > 300 nm). On the other hand, most widespread packaging polymers (cellophane, polystyrene (PS), and poly(ethylene-terephthalate) (PET)) have superior UV-barrier properties showing total absorptivity in the lower wavelength region. Moreover, with the development of large-scale production of PLA, its application in the production of fibers for textiles has gained increasing interest also because its dyeability is similar to that of other polyester materials [2,3]. Therefore, production of PLA having increased UV-Vis barrier properties can be used to design packaging tools suitable for protecting light-sensitive foods and goods or for product UV-shielding fibers for protecting fabrics. Simultaneously, the preparation of dyed PLAs can allow for production of aesthetically connoted plastic items or fibers for textiles or it can be used to produce sutures and implants that can be identified by the naked eye or by using a black light lamp. All these items represent remarkable achievements that can expand the potential applications of PLA. A possible strategy to obtain dyed or UV-protecting polymers is the addition of low molecular weight chromophores to the polymer matrix. However, generally, to extend the service life of polymers where additives are added, it is necessary to increase the affinity between additives and polymer matrices or to immobilize them by the formation of a covalent bond with the polymer backbone or by the use of host–guest systems. The formation of covalent bonds between the additive and polymer chains is probably the most interesting approach because it can prevent migration, volatilization, and possible loss by leaching of the additive. In the case of PLA, this can be carried out by synthesizing the polymer by ring opening polymerization (ROP) of lactide [4]. This is a well-documented polymerization methodology that ensures the production of functionalized end-capped PLA having high and controlled molecular weight. The polymerization process needs the use of a catalyst—generally stannous octoate but other catalysts are described [5,6,7]—and of a co-initiator—generally, an alcohol even though other systems are also reported in the literature [8]. Interestingly, the use of difunctional alcohols as co-initiators produces functionalized end-capped PLA. For example, by this route, PLAs bearing covalently bonded chromophores [9,10,11], UV absorbers [4,12], long alkyl chain [13,14,15], and fluoroalkyl chain [16] are described in the literature. In this paper, 9H-carbazol-ethanol (CA), 9-fluorenyl-methanol (FM), and 2-(4-(2-chloro-4-nitrophenylazo)-N-ethylphenylamino)ethanol (Disperse Red 13, DR) (Figure 1) are used for the first time as co-initiators for the synthesis of end-functionalized PLA.

The three chromophores are selected on the basis of their UV-Vis characteristics with the aim to transfer their optical properties to the biodegradable polymer without changing its main features. FM and CA absorb in the UV-A and UV-B regions and emit by fluorescence in the range between 300 and 400 nm, whereas DR is a commercially available dye with an absorption maximum at about 480 nm in chloroform (in the visible part of the electromagnetic spectrum). The end-functionalized polylactides produced by using CA, FM, or DR as initiators can be considered “macromolecular dyes” (MDs) that can be used “as synthesized” or can be blended with commercial PLA. By this way, dyed PLA can be obtained. The polymerization reaction of L-lactide is initiated by conventional thermal treatment that is carried out in the bulk, but in the case of FM, microwave-assisted polymerization was also used. To the best of our knowledge, chromophores in general and FM in particular have never been used before in the microwave-assisted polymerization of L-lactide. The details and results of this part are reported in a separate section at the end of the main text. All MDs are structurally characterized by size exclusion chromatography (SEC), Fourier transform infrared (FT-IR) spectroscopy, and proton nuclear magnetic resonance (^1^H-NMR) analysis. Thermal properties are evaluated by differential scanning calorimetry (DSC) analysis, and photo-physical features are determined by ultraviolet (UV)-visible absorption (UV-Vis) spectroscopy and fluorescence emission spectroscopy. Finally, migration tests on samples where MDs are mixed with PLA are carried out under different experimental conditions and the results are compared with those obtained for samples obtained by mixing the free chromophores with PLA.

## 2. Materials and Methods

### 2.1. Materials and Reagents

PLA 2002D containing 96% of L-lactide and supplied by NatureWorks^®^, Minnetonka, Minnesota USA having melt flow index (MFI) (2.16 kg/190 C) 4–8 g/10 min, was used as a polymer matrix. Before processing, PLA was dried in a vacuum oven at 110 °C for 12 h. 9H-carbazole-ethanol (CA, Aldrich, St. Louis, Missouri, USA), 9-fluorenyl-methanol (FM, Aldrich; St. Louis, Missouri, USA), 2-[4-(2-chloro-4-nitrophenylazo)-N-ethyl-phenylamino]ethanol (disperse red 13, DR, Aldrich), tin(II) 2-ethylhexanoate or stannous octoate (Sn(Oct)_2_) (Aldrich, St. Louis, Missouri, USA), chloroform (for HPLC, ≥ 99.8 %, contains 0.5–1.0% of ethanol as stabilizer) were used as received. L-lactide (Aldrich, St. Louis, Missouri, USA) was sublimed before use.

### 2.2. Conventional Synthesis of Macromolecular Dyes (MDs)

Sublimed L-lactide (approx. 2 g) was heated in bulk, under nitrogen atmosphere, in a Schlenk tube at 130 °C for 3 h in the presence of Sn(Oct)_2_ (0.5 mol% with respect to L-lactide) and CA, FM, or DR (1, 3, 5, 7, or 10 mol% with respect to L-lactide) to obtain macromolecular dyes. The new products were named MD followed by the acronym of co-initiator and a number corresponding to the percentage of co-initiators used in the synthesis. For example, MD end capped with CA prepared by using 5 mol% of co-initiator was named MDCA5. Analogously, MD end-capped with FM prepared by using 5 mol% of co-initiator was named MDFM5, and MD end-capped with DR prepared by using 5 mol% of co-initiator was named MDDR5. Purified polymers were obtained by dissolving the crude product in chloroform (20 mL), followed by precipitation that was carried out by slow and controlled addition of n-hexane. The precipitate was recovered by filtration with a Büchner funnel (for powdery, higher-molecular weight polylactides) or decantation (viscous liquid, lower-molecular weight polylactides). Products were isolated in good to excellent yields (75%–95%) after drying overnight in a vacuum (approx. 50 Torr) at room temperature. The conversion of L-lactide was evaluated by considering the ratio between its unreacted amount at the end of reaction and its amount in the feed. The amount of unreacted lactide was quantitatively evaluated by ^1^H-NMR by the signal integration of the quartet associated with the CH of residual L-lactide (5.04 ppm) and that of the repetitive unit of MDs (5.16 ppm) (Figure S1 Supplementary Materials). To evaluate the absence of residual free chromophores, ^1^H-NMR analysis of crude products was used. A shift of the methylene protons in the alpha position of hydroxyl group upon formation of the ester bond between the hydroxyl group and PLA chain was observed. The polymerization degree (PD) (the number of lactic acid repeating units in the polymer) was evaluated by calculating the ratio between the integral of the quartet associated with CH of repeating unit of MD (5.16 ppm) and that of the terminal lactic acid unit (4.36 ppm). The conversion of L-lactide to MDs, PD, number average molecular weight (Mn) determined by ^1^N-NMR and by SEC, Mn calculated on the basis of the L-lactide/co-initiator ratio in the feed and dispersity (Đ) calculated as weight average molecular weight (Mw) and Mn ratio (Mw/Mn) evaluated by SEC are reported in Table 1.

### 2.3. Blending of Commercial PLA with MD (PLA/MD5s)

PLA and MD blends were prepared by using a discontinuous mechanical mixer Brabender Plastograph OHG47055 with a chamber of 30 cc. Torque and temperature data were acquired by the Brabender Mixing software WinMix ver. 1.0. Blending was carried out at 180 °C by mixing 20 g of PLA with 200 mg of MD containing about 5 mol% of chromophore (MDFM5, MDCA5, and MDDR5) at 50 rpm for 10 min. The prepared samples were PLA/MDFM5, PLA/MDCA5, and PLA/MDFM5.

### 2.4. Blending of Commercial PLA with Chromophores (PLA/CHROs)

Analogous to the preparation of PLA/MDs, 20 g of PLA was blended with about 10 mg of CA, FM, or DR (the correct amount was evaluated on the basis of the data reported in Table S1, Supplementary Materials) at 180 °C by using a discontinuous mechanical mixer. The prepared samples were PLA/FM, PLA/CA, and PLA/DR.

### 2.5. Migration Test

Tests for solvent extraction resistance were carried out by cutting about 100 mg of films of PLA/CHRO or PLA/MD into pieces of 2 mm × 2 mm that were suspended in 3 mL of EtOH 95% (EtOH/H_2_O 95/5 v/v) at 40 °C for 90 min. Solutions were analyzed by UV-Vis absorption spectroscopy. The percentage of extracted chromophores was calculated on the basis of the total amount of chromophore in the tested specimen.

In the case of PLA/DR and PLA/MDDR5, other migration tests were carried out to check the transfer of the red color from the pristine PLA sample to another polymer film. Accordingly, films of PLA/DR or PLA/MDDR5 were put in contact with PET or styrene butadiene copolymer (SBC) films. To speed up transfer of the color, PLA samples and PET films were hot compressed at 130 °C (lower than the melting temperature of PLA samples and of PET) for 30 min at 1.2 tons. The experiments were repeated at 100 °C or 120 °C for 30 min by using SBC film in place of PET. Other tests were carried out to check the migration of dyes inside the polymer matrix in the melt. Accordingly, film of PLA/CA or PLA/MDCA5 were hot melted at 180 °C in a parallel plate heating press between aluminum and Teflon sheets for 5 min at 1 ton. The hot samples were quenched at −18 °C for 15 min and then analyzed. The same samples were placed again between aluminum and Teflon sheets and were annealed at 110 °C for 16 h in an oven. The film surfaces were characterized by fluorescence emission spectroscopy.

### 2.6. Instruments

Size exclusion chromatography (SEC) analysis was carried out by the Agilent Technologies 1200 Series instrument equipped with an Agilent degasser, an isocratic high performance liquid chromatography (HPLC) pump, an Agilent refractive index (RI) detector, one pre-column PLgel 5 μm guard, and two PLgel MiniMIX-D 5 μm columns conditioned at 35 °C. Chloroform (CHCl_3_) was used as mobile phase at a flow rate of 0.3 mL/min. The system was calibrated with polystyrene standards in a range from 500 to 3 × 10^5^ g/mol. Samples were dissolved in CHCl_3_ (2 mg/mL) and filtered through a 0.20-μm syringe filter before analysis. Mn and Mw were determined using Agilent ChemStation software. 

FT-IR spectra were collected by Fourier Transform Spectrometer Perkin Elmer Spectrum 100.

^1^H-NMR spectra were recorded in CDCl_3_ solutions by means of a Varian VXR 200 spectrometer operating at 200 MHz for 1H.

Differential scanning calorimetry (DSC) experiments were performed using a Perkin Elmer DSC 7 calorimeter equipped with a liquid nitrogen low-temperature apparatus. Previously, the instrument was calibrated by using lead (m.p. 327.46 °C) and indium (m.p. 156.6 °C, Δ*H*_m_ = 28.5 J/g) as references. Thermal scans were carried out on 5–10 mg samples under nitrogen atmosphere using aluminum pans. Samples were analyzed from 40 °C to 220 °C (1st heating) at a heating rate of 20 °C/min and kept at this temperature for 5 min; afterwards, the samples were cooled from 220 °C to 40 °C (1st cooling) at a cooling rate of 20 °C/min and then heated from 40 °C to 200 °C (2nd heating) at a heating rate of 20°C/min. A second set of experiments was carried out on 5–10 mg samples under nitrogen atmosphere (nitrogen flow was 50 mL/min for all experiments) by using a Perkin-Elmer DSC-4000 differential scanning calorimeter thermal analyzer equipped with a 3 stage cooler able to reach −130 °C. Previously, the instrument was calibrated by using indium (m.p. 156.6 °C, Δ*H*_m_ = 28.5 J/g) and zinc (m.p. 419.5 °C). MDs, PLA/CHROs, and PLA/MDs were heated from 20 to 180 °C at 10 °C/min (1st heating), taken at 180° for 2 min, cooled to −90 °C at the same scan rate (1st cooling), taken at −90°C for 2 min, and then heated again to 180 °C at 10 °C/min (2nd heating). Glass transition temperature (*T*_g_), melting temperature (*T*_m_), melting enthalpy (Δ*H*_m_), cold crystallization temperatures (*T*_cc_), cold crystallization enthalpy (Δ*H*_cc_), and percent of crystallinity were determined from the 2nd heating thermogram. Percent of crystallinity was determined on the basis of the following equation: C% = ((Δ*H*_m_ − Δ*H*_cc_)/Δ*H*_m_^0^) × 100, with Δ*H*_m_ = enthalpy associated with the melting process (when a multimodal melting peak is present, its total area is used), Δ*H*_cc_ = enthalpy associated with the cold crystallization process, and Δ*H*_m_^0^ is the enthalpy associated with the melting process of 100% crystalline PLA (93.1 J/g) [17,18].

UV-Visible absorption spectra were recorded with a Perkin-Elmer Lambda 65 spectrophotometer. Spectra were acquired from polymer films or solutions. The concentration of the solutions was chosen in order to maintain absorbance near 1. The concentrations of free chromophores in CHCl_3_ were 2.35 × 10^−5^ M (CA), 4.50 × 10^−5^ M (FM), and 2.35 × 10^−5^ M (DR). The concentrations of free chromophores in EtOH 95% were 2.27 × 10^−5^ M (CA), 3.73 × 10^−5^ M (FM), and 1.58 × 10^−5^ M (DR). Chloroform solutions of MDs were prepared by dissolving a known quantity of solid MD in a known volume of solvent in order to have absorbance near 1. Chloroform solutions of MD had the following compositions: 0.228 mg/mL (MDCA5), 0.116 mg/mL (MDFM5), and 0.110 mg/mL (MDDR5).

Fluorescence emission and excitation spectra were acquired under isotropic excitation with a Perkin Elmer luminescence spectrometer LS55 controlled by FL Winlab software and equipped with the front-surface accessory. Origin 7.5, software by Microcal Origin^®^, was used for analysis of the absorption and emission data. Some fluorescence emission spectra were collected by a FluoroMax4-TCSPC fluorometer with a xenon lamp as the excitation source. Chloroform solutions and polymer films were analyzed. In the case of film, steady-state fluorescence spectra of polymer films were acquired at room temperature under isotropic excitation by placing the solid sample holder at 30° with respect to the incident beam with the aim to focus the excitation light at the front surface of the samples so that fluorescence emission was collected from the same region, minimizing reflected and scattered light. The Fluorescence Excitation-Emission Map (EEM) was collected by a FluoroMax4-TCSPC fluorometer with a xenon lamp as the excitation source. The EEM spectrum was a collection of a series of emission spectra over a range of excitation wavelengths. In this experiment, the EEM spectrum was collected with subsequent scanning emission spectra from 340 to 450 nm at 2-nm increments by varying the excitation wavelength from 230 to 340 nm at 4-nm increments.

## 3. Results and Discussion

### 3.1. Macromolecular Dyes (MDs)

#### 3.1.1. Preparation, Structural Characterization, and Thermal Properties

**Figure 2:**
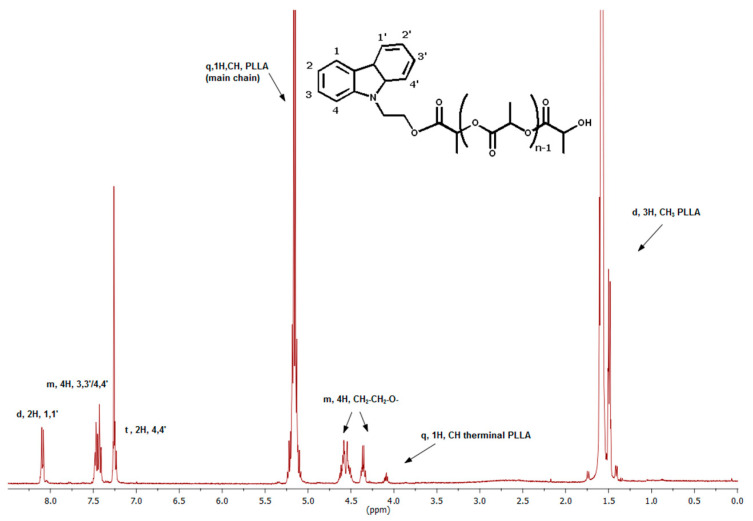
Proton nuclear magnetic resonance ^1^H-NMR spectrum of macromolecular dye (MD) prepared by using 10 mol% of 9H-carbazol-ethanol (CA) (MDCA10) (CDCl_3_).

The preparation of MDs was carried out by ring open polymerization (ROP) of L-lactide catalyzed by Sn(Oct)_2_ in the presence of the co-initiators described in the Introduction. The ratio between the co-initiator and L-lactide was modulated in order to obtain MDs with different molecular weights. Low final molecular weights of MDs were decided upon because this can simplify their characterization and can emphasize the relation between initiator and polymer properties. The formation of PLA by polymerization of L-lactide was verified by comparing the FT-IR spectra of L-lactide and of MDs (Figure S2 and Table S2, Supplementary Materials) [19]. The conversion of L-lactide into MDs was evaluated gravimetrically after purification of the sample, and it was high quantitatively when FM and CA were employed as co-initiators. In the presence of DR, a significantly lower conversion was obtained possibly due to the steric hindrance of the dye hampering the catalyst activity (Table 1). ^1^H-NMR spectra of MDs (Figure 2 for sample MDCA10 and Figures S3 and S4, Supplementary Materials, for samples MDFM5 and MDDR5) were recorded to identify the initiator and to evaluate PD, which is the number of lactic acid repeating units in the polymer (see Section 2.2 and Table 1). The amount of unreacted lactide was quantitatively evaluated by ^1^H-NMR (see Section 2.2 and Figure S1, Supplementary Materials), and about 2–3 wt.% of L-lactide was still present at the end of the reaction because equilibrium between polymerization and depolymerization processes can occur at high temperatures [20,21]. ^1^H-NMR analysis was used also to evaluate the absence of residual free chromophores (see Section 2.2). PD values evaluated by ^1^H-NMR were lower than expected (Table 1) on the basis of conversion but, as discussed later in the text, some side reactions that lowered the PD probably occurred during polymerization.

**Figure 3:**
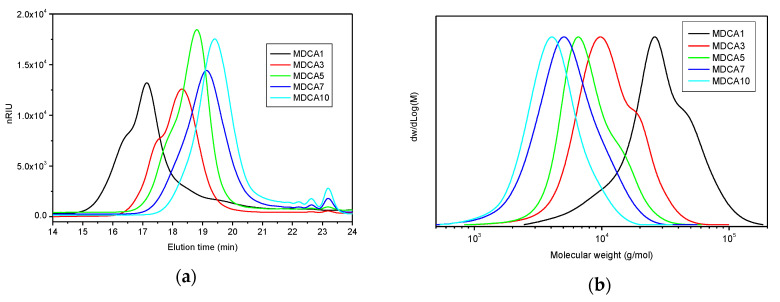
(**a**) Chromatograms collected during size exclusion chromatography (SEC) analysis of MDs prepared by using 1–10 mol% of CA (MDCA1–MDCA10) and (**b**) molecular weight (MW) distribution of samples MDCA1–MDCA10: the curves are normalized to the maximum value.

Purified samples were analyzed by SEC; the chromatogram curves show a main peak associated to elution of MDs and some other peaks eluting at longer retention time (i.e., low molecular weight fractions) (Figure 3a for MDCA and Figure S5 Supplementary Materials for other MDs). Elution of unconverted lactide, oligomers containing two or three lactic acid units, or the unreacted initiator can account for these peaks [22]. The molecular weight distribution curves of the main peak, especially for low molecular weight MDs, are bimodal or multimodal (Figure 3b for MDCA), giving a dispersity higher than 1. The comparison of Mn obtained by SEC with that calculated by considering the PD determined by ^1^H-NMR (Table 1) shows some discrepancies that are probably due to a bias introduced by PS-based SEC calibration. Indeed, differences in hydrodynamic volume and molecular masses between polystyrene and PLA can exist [15]. This discrepancy can be partially compensated by multiplying SEC data for the Mark–Houwink factor of 0.58 [12]. From the SEC evaluation of Mn, a clear trend can be observed, and as expected, molecular weight is higher when the amount of co-initiator (the chromophore) is lower.

The multimodality of the chromatography peaks as well as the presence of species having low molecular weight can be due to multiple factors. For example, in the living polymerization of lactide catalyzed by Sn(Oct)_2_, dispersity can diverge from unity when the reaction is carried out in bulk because of the presence of transesterification and, in particular, intermolecular transesterification reactions that are generally responsible for the enlargement of molecular weight distribution [23,24,25]. By this mechanism, an active chain end (i.e., metal alkoxide terminated) of a growing macromolecule can attack an ester bond internal to another polymer chain in place of the ester bond of L-lactide. By this way, the number of active and dormant chains (i.e., alcohol terminated) is the same but their length is different (Scheme S1, Supplementary Materials). Furthermore, transesterification reactions are found to be more active when a low concentration of alcohol (co-initiator) is used in the ROP and, in this case, the dispersity approached the value of 2. Conversely, at a high concentration of ROH (co-initiator), the role of transesterification was reduced [24,25]. The presence of traces of water can also justify the multimodality of SEC curves because water can act as co-initiator of ROP like alcohols [7]. Multimodal distribution of the MW can also result from solidification of high molecular weight PLAs that formed during the reaction at 130 °C. This can cause inhomogeneous magnetic stirring and poor temperature control that can give side reactions. This cannot occur for low molecular weight PLAs which remain viscous liquids at 130 °C. Finally, in the case of DR, its melting point is close to the reaction temperature and this can prevent complete dissolution and limit homogeneous distribution inside the reaction mixture. Consequently, lower conversion of L-lactide and peaks eluting at high retention time in SEC chromatograms are observed (Figure S5b, Supplementary Materials).

**Figure 4:**
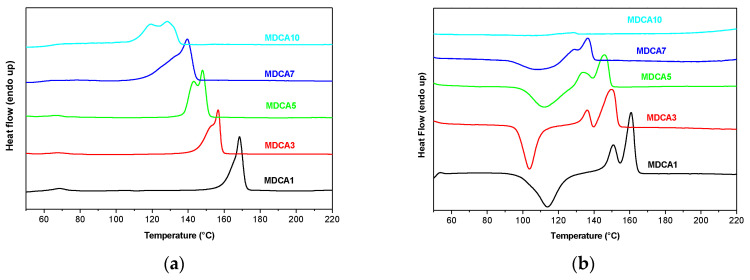
Differential scanning calorimetry (DSC) curves of the first heating (**a**) and second heating (**b**) scans of samples MDCA1–MDCA10: the curves are vertically shifted for clarity.

Thermal properties of MDs were assessed by DSC analysis (Table S3, Supplementary Materials). First and second heating scans were analyzed because different information can be deduced by the analysis of the two curves (MDCA: Figure 4 and Figures S6 and S7, Supplementary Materials, for the other MDs). The absence of a crystallization exotherm peak (related to cold crystallization, *T*_cc_) in the first hating scan provides evidence of the complete crystallinity of the samples (Figure 4a and Figures S6a and S7a, Supplementary Materials). The only exception is sample MDFM1, where an exothermic peak (*T*_cc_) at about 84 °C was observed (Figure S6a, Supplementary Materials). Melting temperature, melting enthalpy, and crystallinity increase with the increase of the molecular weight of MDs and depend on the quantity of chromophores used in the feed (Table S3, Supplementary Materials). The correlation between molecular weight and thermal transitions of PLA was reported for PLA samples having Mn lower than 50–100 Kg/mol and is related to the fact that a better packing can be obtained for longer polymer chains than for shorter ones [17,26,27,28]. Moreover, the presence of large and sterically hindered end groups in low molecular weight PLA can influence *T*_m_ values, and a decrease of *T*_m_ was observed by increasing the dimension of the end group [27]. This can be due to the difficult packaging of the polymer chains in an ordered structure. In some cases, a bimodal melting peak or two separated melting peaks were present. This can be associated with the formation of two crystal forms of PLA: the α and α’ crystals that have different thermal stability and consequently different melting temperatures. The second heating thermograms (Figure 4b and Figures S6b and S7b, Supplementary Materials) of samples having higher molecular weight showed an exothermic peak between 98 and 120 °C due to cold crystallization phenomenon (*T*_cc_) and an endothermic peak due to melting of the polymer at 130–160 °C. The typical PLA cold crystallization process that is absent in the first heating scan can be associated to the formation of irregular crystals that occurs during the heating process. The presence of *T*_cc_ in the second heating scan proves that, despite the low molecular weight of MDs, the samples have no time to crystallize during cooling, giving an amorphous state. The *T*_cc_ value depends on different factors like the molecular weight of the samples and the presence of hindered groups that can slow down the crystallization process. However, *T*_cc_ values of MDs do not follow a predictable evolution probably because of the concurrence of too many parameters. The melting temperatures recorded during the second heating were lower than those of the first heating probably due to the formation of less ordered crystals (the α’ form). No cold crystallization or melting processes were instead observed in the second heating scan of MD10s (prepared by using 10 mol% of chromophores). Probably the presence of the hindered end group and the relatively low molecular weight of MD10s prevent the crystallization process. Finally, in each sample, the enthalpy associated with the cold crystallization process and with the melting was similar, suggesting that crystals formed at *T*_cc_, melted at *T*_m_ (Table S3, Supplementary Materials).

DSC analysis was repeated on the MDs prepared by using 5 mol% of chromophores as co-initiators. The samples were analyzed by using a heating/cooling rate of 10 °C/min in a large temperature range with the aim to detect glass transition temperature (Figure S8 and Table S4, Supplementary Materials). As expected, the use of the slower heating/cooling rate in collecting data determined the shift to lower temperatures of the main thermal transition of the samples; however, despite the thermal shift, the same transitions were observed [29]. Glass transition temperatures were in the expected thermal range considering the molecular weight of the analyzed samples.

#### 3.1.2. Photophysical Properties of MD5s

**Figure 5:**
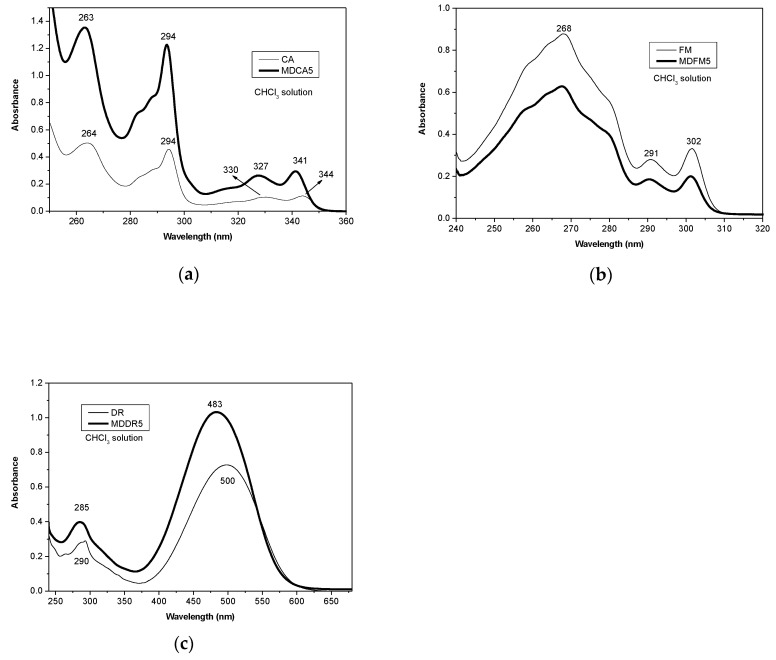
Ultraviolet-visible (UV-vis) absorption spectra in chloroform solution of (**a**) CA and MDCA5, (**b**) 9-fluorenyl-methanol (FM) and MD prepared by using 5 mol% of FM (MDFM5), and (**c**) 2-(4-(2-chloro-4-nitrophenylazo)-N-ethylphenylamino)ethanol (DR) and MD prepared by using 5 mol% of DR (MDDR5): see Section 2.6 for concentrations.

With the aim to highlight potential photophysical differences between free chromophores and MDs, UV-Vis absorption spectra of MD5s (prepared by using 5 mol% of co-initiator) were compared with those of the free co-initiators (Figure 5). All the samples were dissolved in chloroform (see concentration in Section 2.5).

In the UV-Vis absorption spectrum of MDCA5, a very small blue shift of about 3 nm of the band at 344 nm was observed with respect to CA (Figure 5a) [30]. This shift was probably due to the formation of the ester bond between CA and PLA [31]. Instead, the spectra of MDFM5 and FM showed the same absorption maxima (Figure 5b), and these are in agreement with the data reported in the literature [32,33]; no effects of the presence of the PLA chain substituted to the carbon atom in the position 9 were observed. In the case of MDDR5, a blue shit of 17 nm with respect to the absorption band of DR was observed (Figure 5c). This hypsochromic shift could be attributed to the conversion of hydroxyl group in the original dye into the stronger electron-withdrawing ester linkage formed by ROP [34]. By the calibration curves method, the molar extinction coefficient at the absorption maxima of each chromophore was determined (Table S5, Supplementary Materials). These values were used to evaluate the real amount of chromophore present in MDs by measuring the absorbance of chloroform solutions of MDs at known concentrations (Table S1, Supplementary Materials) and by assuming that the molar extinction coefficient of chromophore does not vary after formation of the bond with PLA. From this evaluation, it is observed that, in the case of MDFM5 and MDCA5, about 5 wt.% of chromophores were present in each sample whereas MDDR5 contained about 10 wt.% of DR. All these values were slightly lower than the theoretical ones calculated on the basis of the polymerization feed, and this could be due to a possible loss of chromophores during polymerization and purification.

**Figure 6:**
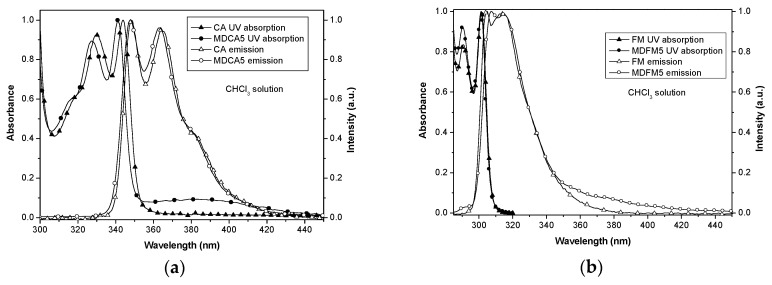
UV-Vis absorption and fluorescence emission spectra: (**a**) CA and MDCA5 in CHCl_3_, λ_exc_ = 290 nm, and (**b**) FM and MDFM5 in CHCl_3_, λ_exc_ = 270 nm. Spectra are normalized to absorption and emission maximum, respectively.

Fluorescence emission spectra of CA and FM were collected from chloroform solution and were compared to the emission spectra of MDCA5 and MDFM5 in chloroform (Figure 6). DR is a non-emitting molecule, and therefore, DR and MDDR5 are not analyses. 

CA and MDCA5 show emission spectra with a vibration structure of the emission band that is very similar to that reported in the literature [31,35,36]. In the spectral region between 300 and 350 nm, the emission spectra of CA and MDCA5 are mirror images of the corresponding absorption spectra. The Stokes shift is 4 nm in the case of CA and 7 nm in the case of MDCA5 (Figure 6a). This difference is probably due to a very small change of carbazoyl geometry due to the presence of PLA chain. Alternatively, different solvation of the steady state and the excited state of CA and MDCA5 associated with the presence of PLA chain could cause the differences in the Stokes shift values. No emission attributable to excimer formation was detected; therefore, carbazolyl moieties in CA or MDCA5 were not interacting with each other. FM and MDFM5 showed similar emission spectra, and both are very similar to that reported for fluorene [37,38], suggesting that the presence of the methanol group or the PLA chain in position 9 of the molecule does not interfere with the emission properties of chromophore. Again, no band attributable to excimer formation was detected (Figure 6b) [33]. 

From the data so far discussed, it is evident that MDs have photophysical properties that are very similar to those of the free chromophores; therefore, their use as co-initiators in the ROP of L-lactide does not modify their photophysical properties that are successfully transferred to the polymer matrix.

### 3.2. Blending of MDs with Commercial PLA

#### 3.2.1. Preparation, Structural Characterization, and Thermal Properties of the Blends

As discussed in the Introduction, the preparation of dyed PLA could be very interesting to obtain new materials having applications in different sectors. One of the main issues associated with mixing of low molecular weight additives to polymer is their migration and leaching especially during the service life of the materials. To limit the loss of additives, the use of molecules having high molecular weight is largely reported and used in the industry. Another key parameter to take into account for the design of polymer additives is the surface energy involved in the polymer–additive interaction. Ideally, the interaction between additives and polymer matrix should be strong to minimize surface energy. Interestingly, the use of MDs as additives satisfies both requirements: MDs are, indeed, chromophores with long PLA chains that increase their molecular weight, lowering their volatility, and the presence of PLA chain can reduce the surface energy of the additives, thus favoring their dispersion. Accordingly, mixing of MDs with commercial PLA can produce stable and homogeneous dispersion of the chromophores into the PLA matrix, limiting also their leaching. Similar methods are also reported in the field of dyeing PLA fibers or tissue, where it is demonstrated that ad hoc modified commercial dyes having a high affinity for the polymer have better dying properties than unmodified dyes [39].

From the previously described MDs, those containing about 5 mol% of chromophores (MD5s) are chosen and mixed to high molecular weight PLA. For comparison, PLA was melt-mixed with free chromophores that were added in an amount equivalent to that present in PLA/MD blends calculated on the basis of the data reported in Table S1 (Supplementary Materials) (the new samples were coded PLA/CHROs, and in particular PLA/CA, PLA/FM, and PLA/DR were prepared). 

SEC analysis of PLA/MDs showed a main elution peak due to PLA, and only in the case of PLA/MDDR5, there was a small peak eluting at a higher retention time, attributable to MDs (Figure S9, Supplementary Materials). DSC analyses of PLA/MDs and of PLA/CHROs showed that *T*_g_ and *T*_m_ of PLA/MDs and of PLA/CHROs were very similar to that of neat PLA (Table 2 and Figure S10, Supplementary Materials). *T*_g_ was about 59 °C, whereas *T*_m_ was about 150 °C, and in all cases, two melting peaks or bimodal melting peaks were detected. *T*_cc_ of PLA/MDs was slightly higher than that of PLA and of PLA/CHROs. Probably, in the case of PLA/MDs, the presence of a low molecular weight PLA end capped with sterically hindered group slowed down the crystallization of the matrix causing the shift of the crystallization process to a higher temperature. Finally, the crystallinity of all the samples was quite low, suggesting that the chains that crystallize at *T*_cc_ melt at *T*_m_.

#### 3.2.2. Photophysical Properties of the Blends

**Figure 7:**
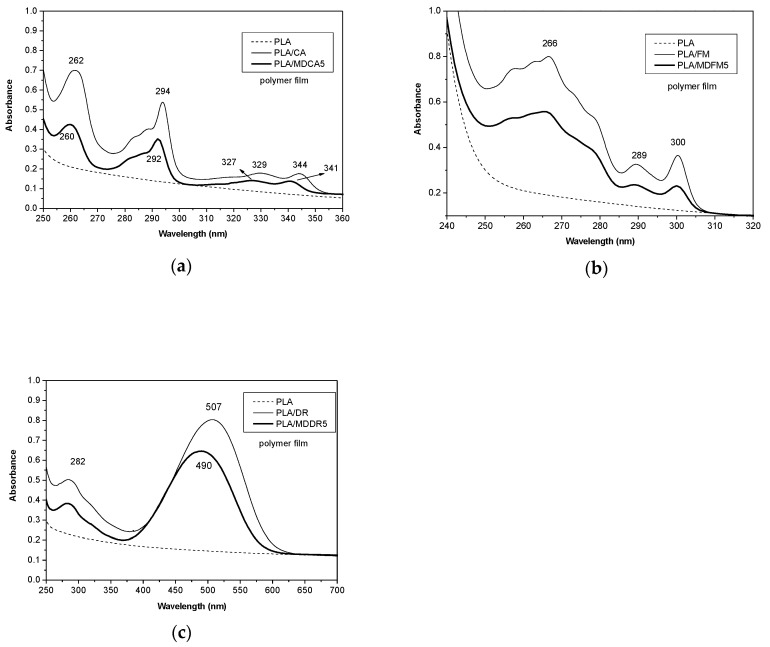
UV-vis absorption spectra were collected from polymeric film: (**a**) poly(lactic acid) (PLA), PLA mixed with CA (PLA/CA), and PLA mixed with MDCA5 (PLA/MDCA5); (**b**) PLA, PLA mixed with FM (PLA/FM), and PLA mixed with MDFM5 (PLA/MDFM5); and (**c**) PLA, PLA mixed with DR (PLA/DR), and PLA mixed with MDDR5 (PLA/MDDR5).

The photophysical properties of PLA/MDs were compared to those of PLA/CHROs with the aim to show the differences between the addition of free chromophores or of MDs to PLA (Figure 7). Photophysical properties of the blends were studied by collecting UV-Vis absorption and fluorescence emission spectra from polymeric films. Absorption spectra and maxima of the samples obtained by mixing CA, MDCA5 (Figure 7a), FM, and MDFM5 (Figure 7b) with PLA were the same as that already observed in the analysis of the chloroform solution of the chromophores and of MDs. Therefore, the presence of PLA does not alter the absorption properties of the pure chromophores and of MDs; in addition, on passing from the solution to the polymer film, no variations of the absorption spectra ware observed. Instead, a bathochromic shift of about 7 nm was observed in the spectra of DR and MDDR5 when they were mixed with PLA (Figure 7c). This effect, that is not due to the formation of aggregates, because the aggregate formation generally causes a hypsochromic shift [40], can be attributed to solvatochromism, a characteristic feature of disperse red derivatives. Generally, on passing from low to high dielectric constant solvents, a red shift (positive solvatochromism) of the main band was observed [41]. The spectrum of DR recorded in EtOH 95% (Table S5, Supplementary Materials) showed an absorption maximum at 507 nm that is 24 nm red shifted with respect to the spectrum recorded in chloroform (Table S5, Supplementary Materials), confirming that solvatochromism can occur also for DR. Although it is generally difficult to compare the photophysical properties of chromophores in solution or embedded in a polymer matrix, the red shift of MDDR5 and of DR mixed with PLA could be attributed to the solvatochromic effect played by the PLA matrix. Indeed, disperse red 1, a chromophore similar to DR, shows a similar bathochromic shift when it is embedded in polymer matrices having different polarity [42].

**Figure 8:**
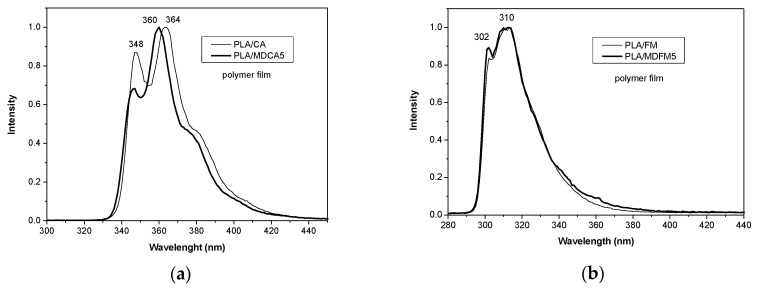
Fluorescence emission spectra of (**a**) PLA/CA and PLA/MDCA5, λ_exc_ = 290 nm, and (**b**) PLA/FM and PLA/MDFM5, λ_exc_ = 270 nm: Spectra are collected from polymer film and are normalized to emission maximum.

Fluorescence emission spectra of the polymer blends were recorded on polymeric films. Again, PLA/MDDR5 and PLA/DR do not show fluorescence emissions, and for this reason, they were not analyzed. The emission spectra of PLA/CHROs and PLA/MDs (Figure 8a,b) were similar to those recorded in solution (Figure 6a,b). However, in the case of the sample containing MDCA, an attenuation of the intensity of the 0-0 band (at about 350 nm) was observed. This self-absorption effect, already observed in the literature, is attributed to the formation of aggregates [31,35,36] that however does not produce excimers since no emission is observed at longer wavelengths. These data suggest that MDs and free chromophores are well dispersed in the amorphous phase of the PLA matrix and that they are not interacting with each other because no aggregate emission was observed. Moreover, the photophysical properties of MDs were completely transferred to PLA films.

**Figure 9:**
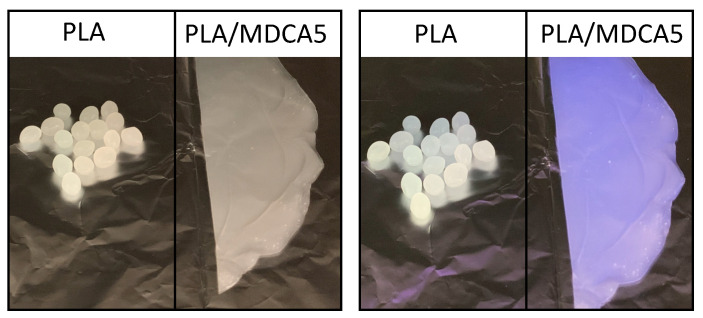
PLA and PLA/MDCA5 under natural light (**left**) and under UV light (λ = 254 nm) (**right**).

Interestingly, PLA/MDCA5 and PLA/MDFM5 have strong absorption bands in the UV-A and UV-B regions whereas they are transparent to visible light, making the film transparent and the dye invisible to the naked eye. This suggests the use of these materials as films for food packaging; indeed, they can protect food from dangerous UV radiation and the packaged goods can be visually observed. Moreover, the UV-A- and UV-B-protecting ability of these MDs can be useful in the production of UV-protecting garments. Furthermore, PLA/MDCA5 and PLA/MDFM5 are fluorescent and, in the case of PLA/MDCA5 its fluorescence emission can be evidenced if it is irradiated with Wood’s lamp emitting at 254 nm (Figure 9). This feature, together with the fact that CA is invisible to the naked eye, can be exploited in the production of track devices for tracing goods along their distribution chain or for anti-counterfeit purposes.

### 3.3. Migration Tests

Two different migration tests were performed: the first set was carried out by suspending the polymer films in a suitable solvent, and the latter was carried out in the solid phase. 

Migration in solution was carried out in EtOH 95% because it is already used to check the migration of antioxidants from PLA nanocomposite films [43] and it is considered a fat simulant in the determination of species that can be released by migration from food contact polymer films such as PLA [22]. By considering that free chromophores and MDs are soluble in this solvent, EtOH 95% is considered a useful solvent for our purposes.

The EtOH solutions, where different PLA samples were suspended, were analyzed by UV-Vis spectroscopy after 90 min of contact. Since chromophores used in this paper show solvatochromic features, UV-Vis spectra of the free chromophores were recorded in EtOH 95% (Table S5, Supplementary Materials, for absorption wavelengths values and molar extinction coefficients at absorption maxima). The results obtained after migration experiments reported in Table 3 (see Section 2.5 for details) evidence that, as expected, a higher quantity of the chromophores was extracted from PLA/CHROs than from PLA/MDs. In the case of films containing MDDR5 and DR, migration tests were carried out also for 3.5 h. The total amount of extracted DR was 35 wt.% with respect to the total amount of DR from PLA/DR and less than 5 wt.% from PLA/MDDR5. This result showed that the few chromophores were lost by PLA/MDDR5 during the first 1.5 h of contact, whereas in the case of PLA/DR, a considerable migration of DR was still going on after 3 h.

**Figure 10:**
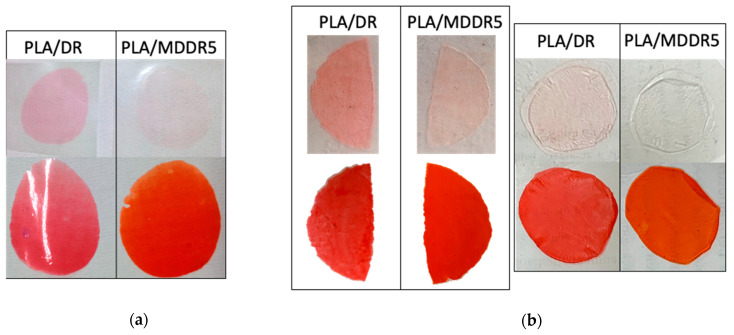
(**a**) Pictures of color transfer from PLA/DR or PLA/MDDR5 to poly(ethylene-terephthalate) (PET) at 130 °C. Upper part of the picture: PET films where the spots of DR (left) and MDDR (right) are present. Lower part of the picture: PLA/DR (left) and PLA/MDDR5 (right) film after the contact with PET. (**b**) Pictures of color transfer from PLA/DR or PLA/MDDR5 to styrene butadiene copolymer (SBC) at 120 °C on the right and at 100 °C on the left. Upper part of the pictures: styrene butadiene copolymer (SBC) films where the spots of DR and MDDR are present. Lower part of the pictures: PLA/DR and PLA/MDDR5 film after contact with SBC (see the text and Section 2.5 for details.).

Taking inspiration from the textile industry, where the leaching of dyes from fabric is a very important issue because it can occur during processing steps and the service life of the garment, we were intrigued by the possibility to qualitatively test the transfer of free chromophores or MDs from PLA films to another polymer. This can be considered an alternative method to evidence the migration of a dye embedded in a polymer matrix. Indeed, only when the chromophore is free to migrate (not covalently bonded to the polymer backbone or immobilized) and its affinity is higher for another polymer than for PLA could we observe the transfer of color from PLA to another material. In particular, we carried out some experiments by putting dyed PLA films in contact with two different polymer films. To better evidence the staining, i.e., the transfer of color from PLA to anther polymer, PLA/DR and PLA/MDDR were used because DR absorbs in the visible portion of the electromagnetic spectrum and can be easily detected. Considering the molecular structure of DR, it is possible to hypothesize its good chemical affinity for polymers having aromatic groups like polystyrene (PS) and poly(ethylene terephthalate) (PET). In this work, PET and styrene-butadiene copolymer (SBC), which is more flexible than pure PS, were used. To speed up the process, the experiment was carried out in a hot press where films of PLA/DR and PLA/MDDR were compressed on PET films at 130 °C for 30 min (Figure 10a). The experiment was repeated by compressing dyed PLA films on SBC films at 100 °C or at 120 °C for 30 min (Figure 10b).

From the pictures reported in Figure 10a,b, it is evident that staining of PET or SBC is observed only for the PLA/DR sample, suggesting that DR has a higher affinity for PET and SBC than for PLA. As expected, MDDR has a lower migration ability than DR. These findings definitely assessed that, once embedded in PLA, MDDR is less susceptible to leaching than DR and that the immobilization of DR in MDDR significantly reduces its migration from the PLA matrix.

**Figure 11:**
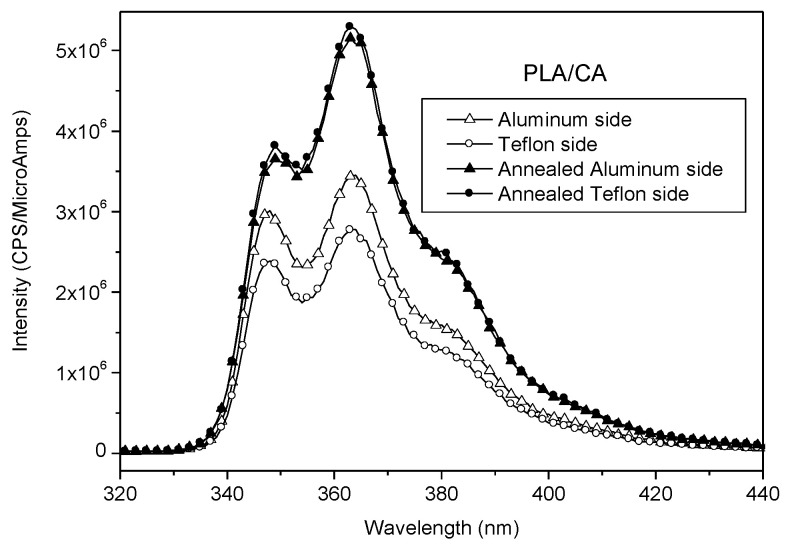
Fluorescence emission spectra collected from PLA/CA before and after annealing from the aluminum and Teflon sides.

**Figure 12:**
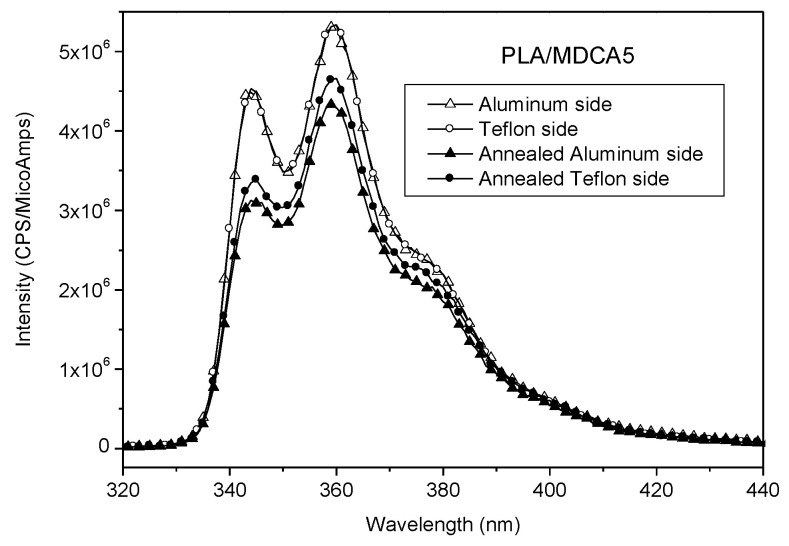
Fluorescence emission spectra collected from PLA/MDCA5 before and after annealing from the aluminum and Teflon sides.

Other migration tests are carried out starting from solid PLA samples taking advantage of the fact that an asymmetric phase distribution of a polymeric blend can be obtained by its compression molding between two surfaces having different polarity like Teflon and aluminum sheet [44]. Generally speaking, enrichment on the surface of one constituent of a polymer blend has important implications in those applications that involve the surface properties like wettability, adhesive interactions, and fouling resistance [45]. Taking inspiration from these observations, films of PLA/CA and PLA/MDCA5 are hot melted at 180 °C between aluminum and Teflon sheets. The surface energy of aluminum is 39.5 mJ/m^2^, that of Teflon is 13.6 mJ/m^2^ [44], and that of PLA is 42 mJ/m^2^ [45]. CA has a polar group, and for this reason, it is expected that its migration is favored towards aluminum rather than towards Teflon that has a lower surface energy. After compression molding between aluminum and Teflon and quenching at low temperature, the two faces of PLA films were analyzed by fluorescence emission. In the case of PLA/CA, the spectrum collected from the aluminum-exposed side was more intense than that recorded from the Teflon side (Figure 11, full symbols); in the case of PLA/MDCA, the spectra recorded from both faces had the same intensity (Figure 12, full symbols). As hypothesized, CA migrates and accumulates on the surface facing to aluminum, but migration occurred only in PLA/CA; indeed, when MDCA was mixed to PLA, no surface segregation of chromophore was observed. The samples just described were annealed at 110 °C for 18 h by maintaining the contact of the film surfaces with aluminum or Teflon. This procedure was performed with the aim to increase the crystallinity of PLA because it is expected that chromophores are dispersed into the amorphous phase of the matrix and an increase of crystallinity can induce chromophores to aggregate or to further accumulate on the surface. The emission spectra collected after annealing (Figure 11 and Figure 12 empty symbols) showed that, in the case of PLA/CA, the fluorescence intensity increased and it was similar on both sides of the film: no excimer emission was detected. The fact that fluorescence intensity was similar on both sides suggested that the increase of crystallinity caused the accumulation of chromophore on both sides independently from the different polarity of the surfaces in contact with PLA. In the case of PLA/MDCA, the fluorescence intensity was slightly lower after annealing than before; probably in this case, no migration was possible because of the presence of the PLA chain bonded to the chromophore that can be involved in the formation of crystals within the PLA chains constituting the matrix. Finally, it can be observed that the relative intensity of the band at about 350 and 360 nm was changed by the annealing process (Table S6, Supplementary Materials). As previously discussed, the lower intensity of the band at about 350 nm with respect to the band at about 360 nm can be associated with the presence of aggregates. Interestingly, in PLA/CA and PLA/MDCA5 after annealing, the ratio between the band intensity at 350 and 360 nm was lower than before annealing (see Figure 11 and Table S6, Supplementary Materials), and this occurred on both sides. This suggests that, after annealing, the segregation of chromophores induced by the increase of crystallinity of PLA, caused also the formation of aggregates even if in the case of PLA/MDCA5 (see Table S6, Supplementary Materials) the effect was less evident.

The results achieved in this paper could be interesting also in the field of fabrics or textile where fibers of PLA are used. This could be particularly important because PLA fibers are emerging as an eco-friendly alternative to conventional PET fibers. The affinity between the fiber and the dye is one of the key parameters that control dying of the PLA fibers. Disperse dyes are indeed “absorbed” or better exhausted by the fibers, and the process could be considered the dissolution of disperse dyes in a solid solvent (the polymer fiber) [2,3,39]. This can be facilitated, as proven here, if the chromophores contain a long PLA chain. Moreover, the “thermo migration” and the “storage migration” [46,47] of dyes can also be in part limited by using dyes having high molecular weight or high affinity for the fibers. These undesired processes occur during the production of dyed and during their storage. By this phenomenon, the dye accumulates on the surface of the fiber and can be lost by sublimation, can be removed during subsequent washing, or can stain adjacent fabrics, causing an overall depletion of the appearance of the fibers.

### 3.4. Microwave Assisted Polymerization of L-lactide in the Presence of FM as Co-initiator

In the case of FM in parallel to the classical ROP reaction carried out, heating the reaction mixture at 130 °C, a series of microwave-assisted polymerization reactions were performed. Microwaves were considered an efficient energy source for the synthesis of polymers, with an impressive potential in terms of time and energy economy and sometimes capable of inducing specific nonthermal effects [48,49,50,51]. By this way, microwave-assisted polymerization of L-lactide can proceed faster, with high purity and consequently with high yield if compared with the classical lactide polymerization. Finally, lactide, lactic acid, and its oligomer are polar molecules so they can absorb microwave energy, thus increasing the temperature of the reaction [19]. In the literature, there are few examples that use a co-initiator in the preparation of PLA by microwave irradiation [52,53,54], and to the best of our knowledge, FM that is a chromophore, is for the first time used as a co-initiator in the ROP of L-lactide under microwave irradiation. Experimental details for the preparation of macromolecular dyes end capped with FM prepared by microwave-assisted polymerization of L-lactide (MDFMmws) are reported in the Supplementary Materials. The conversion of L-lactide, PD, Mn evaluated by ^1^H-NMR, Mn and Đ evaluated by SEC analysis, and Mn calculated on the basis of L-lactide/initiator in the feed are reported in Table 4.

From the data reported in Table 4, it can be observed that the microwave-assisted polymerization of L-lactide initiated by FM generally gives a higher degree of polymerization and consequently higher molecular weight than the classical thermal polymerization of L-lactide. However, for the samples prepared using a higher co-initiator/L-lactide ratio, a lower conversion of the monomer with respect to the classical ROP is observed. Notably, these data are obtained by using a quite shorter reaction time than that used in the conventional thermal reaction; thus, microwave-assisted polymerization can be considered an energy-saving process with respect to other polymerization procedures. 

**Figure 13:**
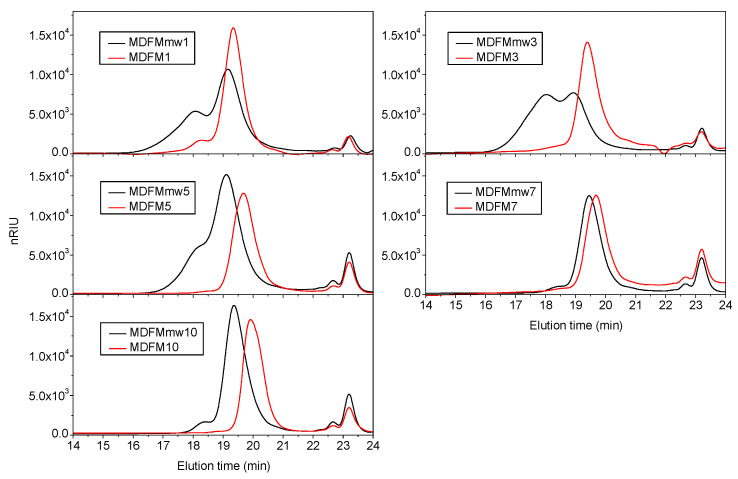
Comparison between chromatograms collected during SEC analysis of the macromolecular dyes samples prepared by microwave-assisted polymerization of L-lactide and by using 1–10 mol% of FM (MDFMmw1–MDFMmw10) and MDFM1–MDFM10.

The molecular weight of the sample is determined by SEC analysis. Chromatographic curves (Figure 13) evidence a main peak eluting between 17 and 20 min due to MDFMmws and some other peaks eluting at a longer retention time. As previously discussed, these peaks can be associated with the presence of an unreacted monomer, the co-initiator, or oligomers. Comparison between the chromatographic curves of MDFMmws with those of MDFMs prepared by the classical thermal polymerization (Figure 13) shows that the molecular weight of MDFMmws is generally higher than that of MDFMs. However, in some samples, especially those prepared by using a low concentration of FM with respect to L-lactide (MDFMmw1 and 3), the main elution peak is bimodal. From the corresponding profile, it can be hypothesized that two families of macromolecules are present in the samples MDFMmw1 and MDFMmw3; however, their formation and their nature are not certain. Although it is difficult to hypothesize the origin of these two populations, the literature suggests that transesterification reactions can occur during polymerization [48]. Transesterification may occur by inter- and intramolecular mechanisms giving, in the first case, an enlargement of molecular weight distribution curves and, in the second case, cyclic polymers. Moreover, for longer reaction times, some authors report also that some degradation phenomena can occur [47]. Alternatively, the two polymer families can be formed independently because of the occurrence of two polymerization processes initiated by different co-initiators, such as FM and water.

**Figure 14:**
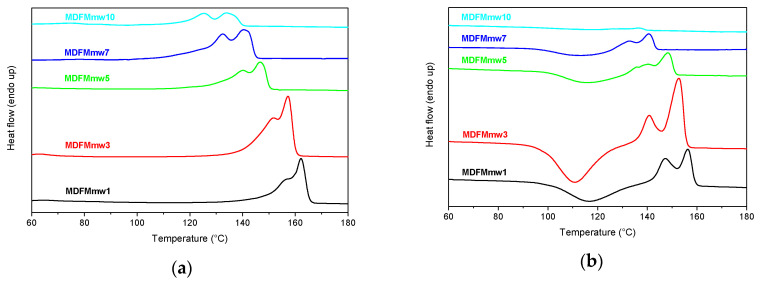
DSC curves of first heating (**a**) and second heating (**b**) scans of samples MDFMmw1–MDFMmw10: Heating and cooling scans were carried out at 20 °C/min. The curves are vertically shifted for clarity.

MDFMmws were also analyzed by DSC (Figure 14; Table S7, Supplementary Materials). The samples showed thermal properties that are similar to those collected for the other MDs. As in the MDFMs samples, also in this case, melting temperatures shifted to higher temperatures with the increase of molecular weight. Two crystalline forms were generally present, both in the first and second heating scans. During the second heating scan, all samples showed a cold crystallization process; the only exception was MDFMmw10. Finally, the enthalpies associated with this process and the melting one were similar, suggesting that the fraction of the sample that crystallizes at *T*_cc_ melted at *T*_m_.

## 4. Conclusions

MDs have been prepared by chromophore-initiated ring opening polymerization of L-lactide catalyzed by Sn(Oct)_2_. Three different chromophores (i.e., CA, FM, and DR) were tested, and variable co-initiator/L-lactide ratios were investigated to tune the molecular weight of MDs. This study reports also for the first time the microwave-assisted polymerization of L-lactide with a chromophore (FM) as a successful example of energy-saving process.

Generally, higher conversion and better control of molecular weight distribution were obtained by using CA and FM in a low concentration with respect to L-lactide. Interestingly, MDs showed thermal properties dependent on molecular weight and photophysical characteristics that are similar to those of free chromophores used as co-initiators. This last feature suggests that it may be possible to use the optical response of MDs in different applications. 

Thanks to the presence of PLA chains bonded to chromophores, the affinity of MDs for PLA is increased and the volatility of chromophores is reduced. Therefore, a dyed PLA (PLA/MD) was successfully obtained by blending MDs with PLA. Interestingly, the photophysical properties of PLA/MDs are very similar to those of free chromophores and of MDs. Moreover, they are characterized by thermal properties similar to that of the PLA matrix, confirming that these materials are suitable for packaging applications.

Migration tests carried out by using different methods definitely confirmed that MDs are significantly less released from PLA films than free chromophores. This was strong evidence that the immobilization of chromophores in MDs significantly reduces their migration from the PLA matrix. Moreover, in the case of PLA/MDCA, a variation of the vibrational structure of the emission spectrum was observed after annealing. In this case, the material has the ability to respond to external stimuli and it can be considered thermo-responsible. The optical response, indeed, may be changed simply by heating the material, and in this way, it could be possible to visualize if it has been subject to unwanted heating processes. 

Another interesting remark emerging from the data discussed in the paper deals with the photophysical properties of MDCA and MDFM. Indeed, these two MDs absorb UV-A and UV-B but are transparent to visible light. This makes the films transparent and the dye invisible to the naked eye, suggesting their use in the production of films for food packaging because they can protect food from dangerous UV radiation and are transparent. In addition, these materials may be used for the production of UV-protecting garments. Furthermore, it may be possible to use the fluorescent response of MDCA and MDFM in anti-counterfeit applications.

Finally, all data so far discussed concern PLA films; however, the results achieved in this paper could be interesting also in the field of fabrics or textiles where fibers of PLA are used because our macromolecular dyes can effectively limit the problem of “storage migration”.

## Figures and Tables

**Table 1:** Conversion of L-lactide to Macromolecular Dyes (MDs), polymerization degree (PD), number average molecular weight (Mn) determined by proton nuclear magnetic resonance ^1^H-NMR and by size exclusion chromatography (SEC), Mn calculated on the basis of L-lactide/initiator in the feed, and dispersity (Đ = Mw/Mn) determined by SEC.

Entry	Conversion (%) ^1^	PD (NMR) ^2^	Mn (NMR) (g/mol) ^3^	Mn (SEC) (g/mol) ^4^	Mn (CAL) (g/mol) ^5^	Đ (SEC) ^6^
MDCA1	93	41	3166	12,702	13,616	1.5
MDCA3	99	26	2085	5452	7346	1.4
MDCA5	99	28	2229	4002	3065	1.3
MDCA7	91	17	1436	2633	2085	1.4
MDCA10	88	13	1148	2088	1480	1.3
MDFM1	92	9	845	2262	13,457	1.2
MDFM3	91	10	917	2146	4568	1.1
MDFM5	99	26	2142	3822	3050	1.2
MDFM7	99	19	1566	1682	2234	1.1
MDFM10	99	4	485	1334	1623	1.1
MDDR1	84	6	781	2320	12,456	1.2
MDDR3	85	10	1069	2407	4432	1.1
MDDR5	82	28	2367	2204	2713	1.1
MDDR7	82	7	853	1218	2037	1.1
MDDR10	82	4	637	754	1531	1.1

^1^ Conversion of lactide ((LA_0_ − LA)/LA_0_) × 100, where LA_0_ is the amount of L-lactide in the feed and LA is the amount of unreacted L-lactide at the end of reaction. The amount of unreacted L-lactide is quantitatively evaluated by ^1^H-NMR by the signal integration of the quartet associated with the CH of residual L-lactide (5.04 ppm) and that of the repetitive unit of MDs (5.16 ppm). ^2^ Determined by calculating the ratio between integrals of CH quartet in the repetitive unit (5.16 ppm) and terminal CH quartet (4.36 ppm) of MDs; values are approximated to the nearest whole number. ^3^ Mn determined by ^1^H-NMR analysis (PD × 72 (lactic acid molecular weight) + M_i_, where M_i_ is the molecular weight of the initiator. ^4^ Mn determined by SEC analysis and corrected by multiplying 0.58 [12]. ^5^ Theoretically calculated Mn = 144.137 (molecular weight of lactide) × (LA_0_/I_0_) × conversion + M_i_, where I_0_ is the mole of co-initiator in the feed. ^6^ Dispersity (Đ = Mw/Mn) determined by SEC analysis.

**Table 2:** Differential scanning calorimetry (DSC) data ^1^ of poly(lactic acid) (PLA), PLA mix with free chromophores (PLA/CHROs), and PLA mixed with MDs (PLA/MDs).

Sample	*T*_g_ (°C)	*T*_cc_ (°C)	Δ*H*_cc_ (J/g)	*T*_m_ (°C)	Δ*H*_m_ (J/g)	Cryst.^2^ (%)
PLA	57.5	113.7	−2.2	149.5	3.4	3.7
PLA/CA	58.9	114.2	−31.6	149.9 156.2	32.9	1.5
PLA/FM	59.1	114.9	−32.6	150.2 156.6	32.4	−
PLA/DR	58.3	131.0	−13.7	153.3	17.6	4.0
PLA/MDCA5	58.8	119.5	−32.9	151.2	32.7	−
PLA/MDFM5	58.8	119.5	−33.7	152.0	32.1	−
PLA/MDDR5	59.2	120.1	−30.9	151.3	37.6	7.2

^1^ Heating and cooling scans were carried out at 10 °C/min. ^2^ Crystallinity was evaluated on the 2nd heating scan by considering the enthalpy associated with the melting process of 100% crystalline PLA = 93.1 J/g [17,18].

**Table 3:** Amount ^1^ of chromophore extracted from PLA mixed with free chromophores (PLA/CHROs) and PLA/MDs.

Chromophore	PLA/CHROs (wt.%)	PLA/MDs (wt.%)
CA	40	9
FM	74	8
DR	28	4

^1^ Evaluated as weight percent of extracted chromophore with respect to its amount in the film.

**Table 4:** Conversion of L-lactide to MDs, PD, Mn determined by ^1^H-NMR and by SEC, Mn calculated on the basis of L-lactide/initiator in the feed, and Đ determined by SEC.^1.^

Entry	Conversion (%) ^2^	PD (NMR) ^3^	Mn (NMR) (g/mol) ^4^	Mn (SEC) (g/mol) ^5^	Mn (CAL) (g/mol) ^6^	Đ (SEC) ^7^
MDFMmw1	93	52	3940	3321	13,457	1.7
MDFMmw3	90	38	2932	4389	4568	1.5
MDFMmw5	88	29	2284	2981	3050	1.4
MDFMmw7	77	27	2140	2006	2234	1.2
MDFMmw10	79	16	1348	2105	1623	1.2
MDFM1	92	9	845	2262	13,457	1.2
MDFM3	91	10	917	2146	4568	1.1
MDFM5	99	26	2142	3822	3050	1.2
MDFM7	99	19	1566	1682	2234	1.1
MDFM10	99	4	485	1334	1623	1.1

^1^ MDFMs data are reported for comparison. ^2^ Conversion of lactide ((LA_0_ − LA)/LA_0_) × 100, where LA_0_ is the amount of lactide in the feed and LA is the amount of unreacted lactide at the end of reaction. The amount of unreacted lactide is quantitatively evaluated by ^1^H-NMR by signal integration of the quartet associated with the CH of residual L-lactide (5.04 ppm) and that of the repetitive unit of MDs (5.16 ppm). ^3^ Determined by calculating the ratio between integrals of CH quartet in the repetitive unit (5.16 ppm) and terminal CH quartet (4.36 ppm) of MDs; values are approximated to the nearest whole number. ^4^ Mn determined by ^1^H-NMR analysis (PD × 72 (lactic acid molecular weight) + M_i_), where M_i_ is the molecular weight of initiator. ^5^ Mn determined by SEC analysis and corrected by multiplying 0.58 [12]. ^6^ Theoretically calculated Mn = 144.137 (molecular weight of lactide) × LA_0_/I_0_ × conversion + M_i_, where I_0_ is the mole of initiator in the feed. ^7^ Đ determined by SEC.

## Supplementary Materials

The following are available online at https://www.mdpi.com/2073-4360/12/9/1979/s1, Figure S1. ^1^H-NMR of a crude polymerization product and enlargement on the CH quartet signals of L-lactide centered at 5.04 ppm and of MD main chain centered at 5.16 ppm, monitored to assess lactide conversion; Figure S2. FT-IR spectra of L-lactide (top) and polylactide (bottom); Figure S3. ^1^H-NMR spectrum of MDFM5 (CDCl_3_); Figure S4. ^1^H-NMR spectrum of MDDR5 (CDCl_3_); Figure S5. Chromatograms collected during SEC analysis of samples a) MDFM1–MDFM10 and b) MDDR1–MDDR7; Figure S6. DSC curves of the first heating (a) and second heating (b) scans of samples MDFM1–MDFM10: heating and cooling scans were carried out at 20 °C/min. The curves are vertically shifted for clarity; Figure S7. DSC curves of the first heating (a) and second heating (b) scans of samples MDDR1–MDDR7: heating and cooling scans were carried out at 20 °C/min. The curves are vertically shifted for clarity; Figure S8. DSC curves of the second heating scan of samples MDCA5, MDFM5, and MDDR5: heating and cooling scans were carried out at 10 °C/min. The curves are vertically shifted for clarity; Figure S9. Chromatograms collected during SEC analysis of PLA/MDCA5, PLA/MDFM5, and PLA/MDDR5; Figure S10. DSC curves (second heating scan) of PLA, PLA/MDCA5, PLA/MDFM5, and PLA/MDDR5 (a) and of PLA, PLA/CA, PLA/FM, and PLA/DR (b): the curves are vertically shifted for clarity. Heating and cooling scans were carried out at 10 °C/min. Table S1. Absorption maxima of MDCA5, MDFM5, and MDDR5 in CHCl_3_ and calculated amount of chromophore in MDs in comparison with the theoretical value in the feed; Table S2. Main FT-IR band of L-lactide and PLA; Table S3. DSC data of MDs; Table S4. DSC data of MDs prepared by using 5 mol% of an initiator: second heating scan; Table S5. Absorption maxima and corresponding molar extinction coefficients of CA, FM, and DR in CHCl_3_ and in EtOH 95%; Table S6. The ratio between the emission intensity of the band at 350 and 360 nm of the spectra of PLA/CA and PLA/MDCA5; Table S7. DSC data of MDFMmws. Scheme S1. (1) Propagation reaction: the active chain attacks the ester bond of lactone, where the opposite reaction is the depolymerization, and (2) intermolecular transfer reaction: the active chain attacks an ester bond internal to another polymer chain, where this chain can be active (metal alkoxide terminated) or dormant (alcohol terminated): Microwave-assisted synthesis of Macromolecular Dyes (MD) by using FM as a co-initiator.
