# Supplementary material for: Macromolecular Dyes by Chromophore-Initiated Ring Opening Polymerization of L-Lactide
Source: Polymers (Basel). 2020 Aug 31;12(9):1979. doi: 10.3390/polym12091979 (PMC7564406; doi:10.3390/polym12091979)
Supplement: Supplementary file 1 [file polymers-12-01979-s001.pdf]

Supplementary Materials

Macromolecular dyes by chromophore-initiated ring opening polymerization of L-lactide

Francesca Cicogna [1,*], Guido Giachi [2], Luca Rosi [2], Elisa Passaglia [1], Serena Coiai [1], Roberto Spiniello [1], Federico Prescimone [3,4] and Marco Frediani [2]

1 Consiglio Nazionale delle Ricerche Istituto di Chimica dei Composti OrganoMetallici, Sede Secondaria di Pisa (CNR ICCOM-SS-Pisa), 56124 Pisa, Italy; francesca.cicogna@pi.iccom.cnr.it (F.C.); elisa.passaglia@pi.iccom.cnr.it (E.P.); serena.coiai@pi.iccom.cnr.it (S.C.); roberto.spiniello@pi.iccom.cnr.it (R.S.)

2 Dipartimento di Chimica "U. Schiff" Università degli Studi di Firenze, 50019 Sesto Fiorentino, Italy.; guidogiachi@gmail.com (G.G.); luca.rosi@unifi.it (L.R.); marco.frediani@unifi.it (M.F.)

3 Dipartimento di Chimica e Chimica Industriale Università di Pisa, 56126 Pisa, Italy; federico.prescimone@bo.ismn.cnr.it

4 Consiglio Nazionale delle Ricerche Istituto per lo Studio dei Materiali Nanostrutturati Sede Secondaria di Bologna, (CNR ISMN-SS-Bologna CNR), 40129 Bologna, Italy

* Correspondence: francesca.cicogna@pi.iccom.cnr.it; Tel.: +39-050-31523393

Table S1. UV-Vis absorption maxima of MDCA5, MDFM5 and MDDR5 in $CHCl_3$ and calculated amount of chromophore in MDs in comparison with theoretical value in the feed

Sample	λ_{max} (nm)	A_{max}	Chromophore in MDs (wt.%)	
			Calculated[1]	Theoretical[2]
MDCA5	344	0.300	4.7	7.4
	330	0.284		
	294	1.248		
	264	1.375		
MDFM5	301	0.185	4.7	6.8
	291	0.171		
	268	0.613		
MDDR5	483	1.03	10.6	12.1

[1] These values are calculated on the basis of absorbance determined by collecting UV spectra on MDs chloroform solution (see the experimental part for concentration) and the molar extinction coefficients reported in Table S5. [2] These values are calculated transforming the percent by mol used in the polymerization reaction (i.e. 5 mol% of the chromophore with respect to L-lactide for these samples), in percent by weight considering also the molecular weight of chromophores.

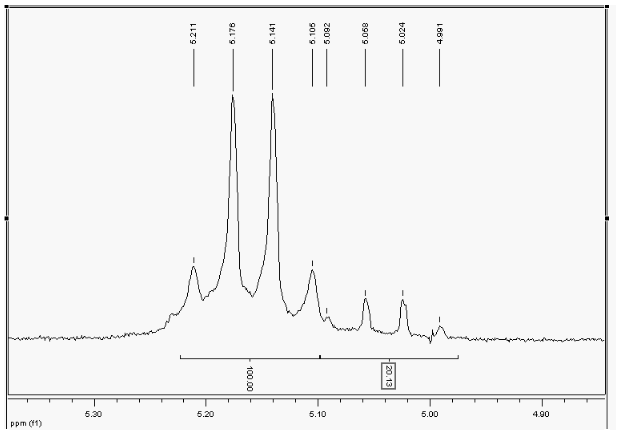

Figure S1. ^{1}H-NMR of a crude polymerization product, enlargement on the CH quartet signals of L-lactide centered at 5.04 ppm and of MD main chain centered at 5.16 ppm, monitored to assess lactide conversion.

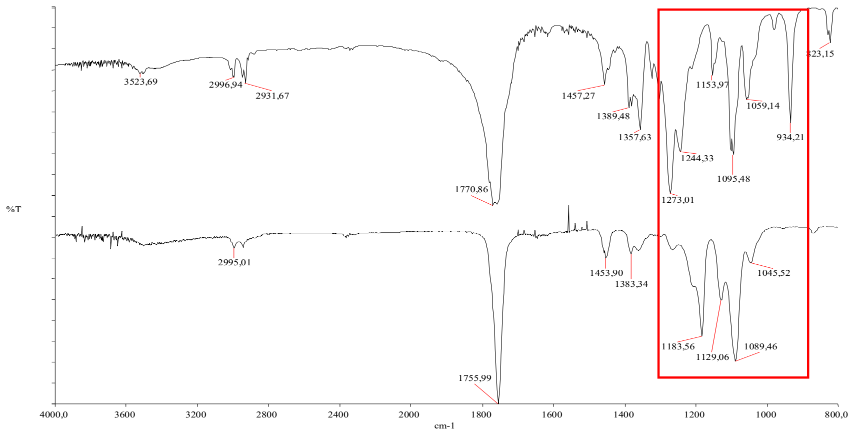

Figure S2. FT-IR spectra of L-lactide (top) and polylactide (bottom).

Table S2. Main FT-IR band of L-lactide and PLA

Vibration	L-lactide	PLA
Stretching C=O	1770 (cyclic lactone)	1755 (aliphatic ester)
Stretching C-O-C asym.	1273 (cyclic lactone)	1183 (aliphatic chain)
Stretching C-O-C sym.	1095 (cyclic lactone)	1089 (aliphatic chain)
COO ring breathing	934 (cyclic lactone)	-

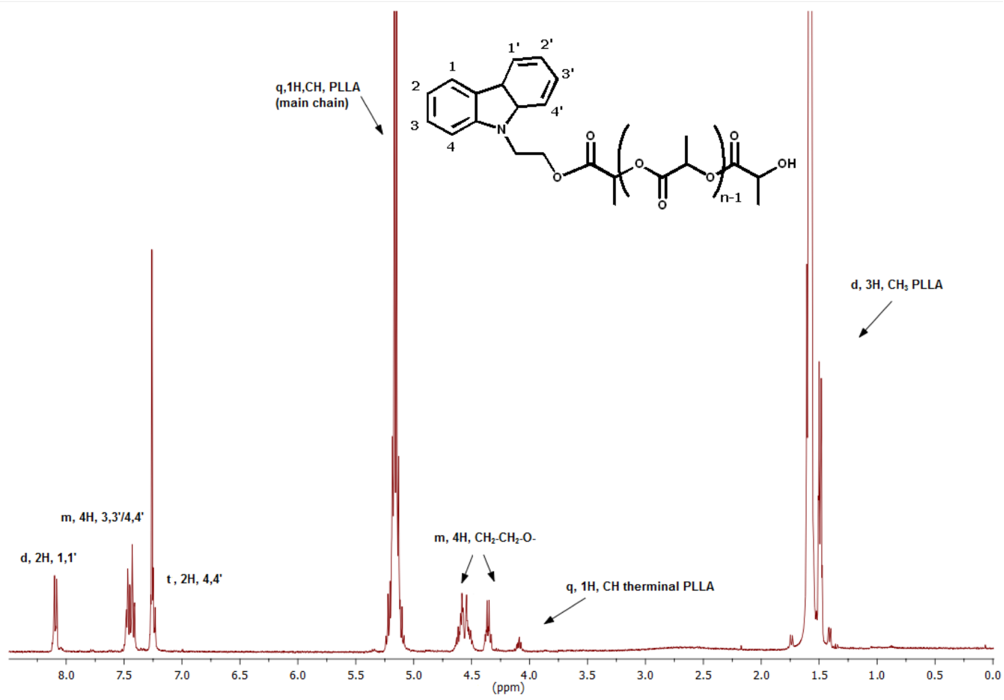

Figure S3. ^{1}H-NMR spectrum of MDFM5 ($CDCl_3$)

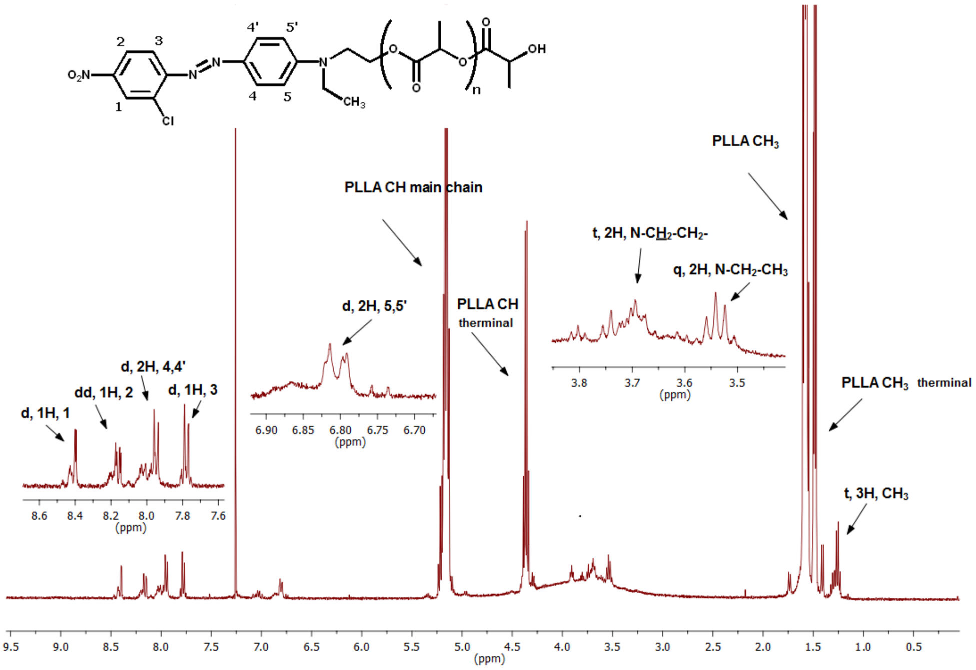

Figure S4. ^{1}H-NMR spectrum of MDFM5 ($CDCl_3$).

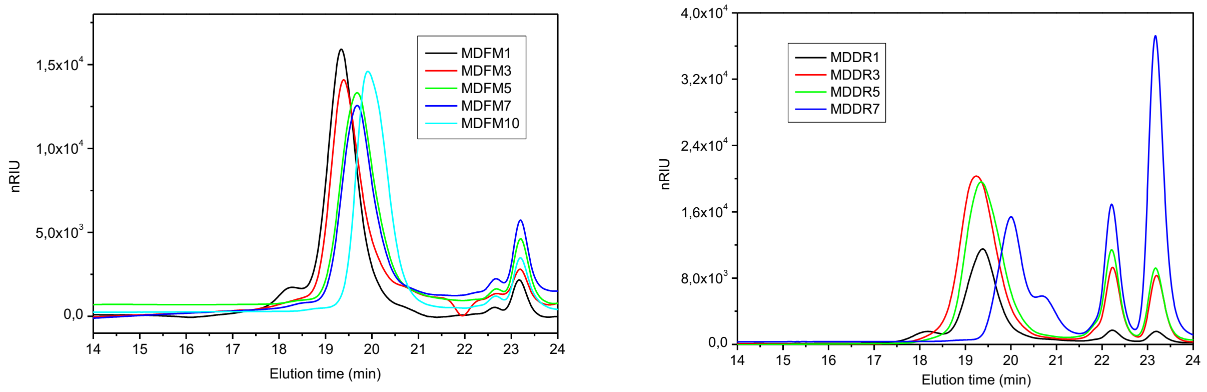

(**a**) (**b**)

Figure S5. Chromatograms collected during SEC analysis of the samples (a) MDFM1-MDFM10 and (b) MDDR1-MDDR7

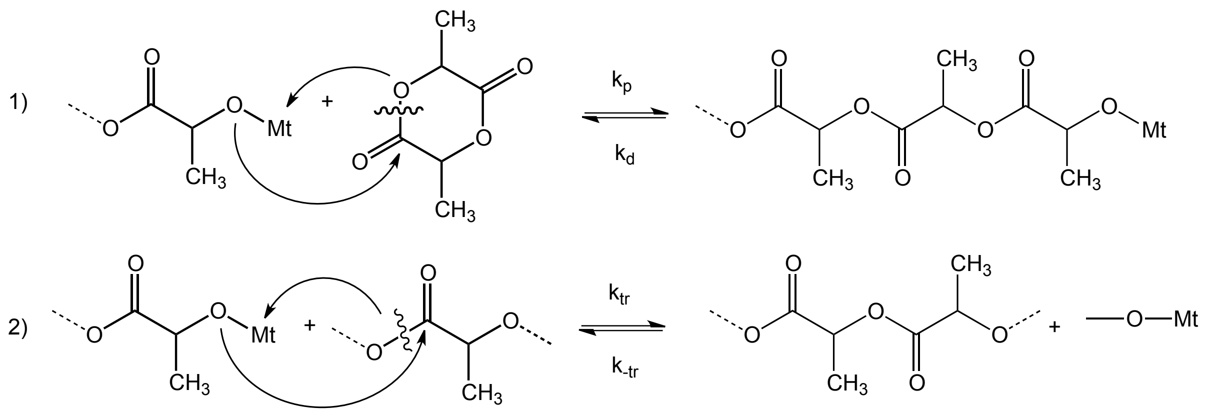

Scheme S1. 1) Propagation reaction: active chain attacks the ester bond of lactone, the opposite reaction is the depolymerization; 2) intermolecular transfer reaction: active chain attacks an ester bond internal to another polymer chain, this chain can be active (metal alkoxide terminated) or dormant (alcohol terminated).

Table S3. DSC data of MDs[1]

Sample	First heating			Second heating			
	T_m (°C)	ΔH_m (J/g)	Crystallinity (%)[2]	T_{cc} (°C)	ΔH_{cc} (J/g)	T_m (°C)	ΔH_m (J/g)
MDCA1	168.5	55.7	60.0	113.0	-41.7	160.5<br>150.0	19.3<br>7.0
MDCA3	156.7	66.8	71.8	103.0	-40.0	149.0<br>136.0	30.8<br>10.1
MDCA5	148.0<br>143.3	38.7	41.6	112.0	-16.9	145.0<br>133.0	15.8<br>-
MDCA7	139.5	48.3	52.0	108.0	-8.5	136.0<br>128.0	8.6<br>-
MDCA10	128.5<br>119.2	31.0	33.3	105.0	-1.2	129.0	0.5
MDFM1	157.6	52.5	56.5	98	-39.1	155.0<br>147.0	44.4
MDFM3	147.5<br>145.0	58.3	62.7	101	-35.1	146.0<br>133.0	39.4
MDFM5	138.5	55.4	59.6	106	-25.6	141.0<br>129.0	26.8
MDFM7	139.5<br>132.0	48.5	52.1	102	-25.0	134.0<br>123.0	26.3
MDFM10	107.8<br>116.1	38.2	41.1	-	-	-	-
MDDR1	154.4<br>148.9	76.5	82.2	98	-25.2	151.0	53.8
MDDR3	139.3	58.7	63.1	102	-33.0	137.0<br>128.0	37.9
MDDR5	132.5	77.0	82.8	105	-30.1	134.0<br>126.0	30.9
MDDR7	89.4	24.3	-	-	-	-	-
MDDR10	n.d	n.d	n.d	n.d	n.d	n.d	n.d

[1] Heating and cooling scan was carried out at 20°C/min. [2] Crystallinity was evaluated on the first heating scan and considering ΔH_m (PLA100%) = 93.1 J/g [Ref 17 and Ref 18 main text].

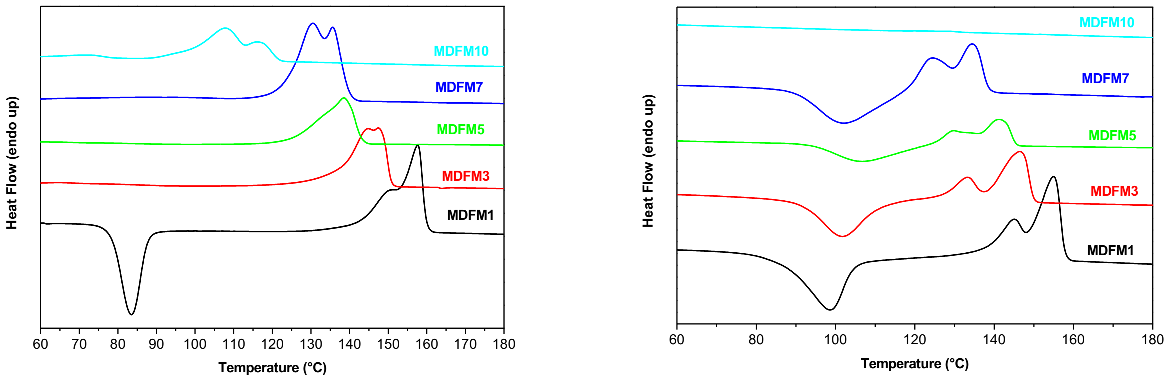

(**a**) (**b**)

Figure S6. DSC curves of first heating (a) and second heating (b) scan of samples MDFM1-MDFM10. Heating and cooling scans were carried out at 20°C/min. The curves were vertically shifted for clarity.

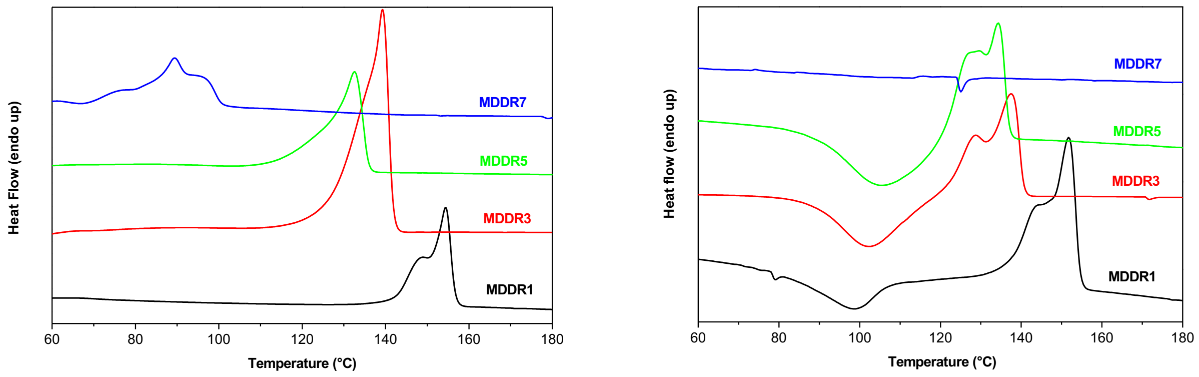

(**a**) (**b**)

Figure S7. DSC curves of first heating (a) and second heating (b) scan of samples MDDR1-MDDR7. Heating and cooling scans were carried out at 20°C/min. The curves were vertically shifted for clarity.

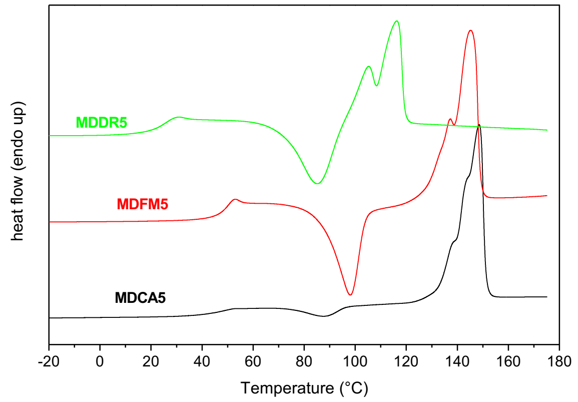

Figure S8. DSC curves of the second heating scan of samples MDCA5, MDFM5 and MDDR5. Heating and cooling scans were carried out at 10°C/min. The curves are vertically shifted for clarity.

Table S4. DSC data of MDs prepared by using 5 mol% of initiator, second heating scan[a]

Sample	T_g (°C)	T_{cc}[b] (°C)	ΔH_{cc} (J/g)	T_m[b] (°C)	ΔH_m (J/g)	Crystallinity[b] (%)
MDCA5b	44.6	87.9	-4.8	148.5	56.5	56
MDFM5b	47.8	98.0	-28.3	137.0<br>145.3	49.6	23
MDDR5b	22.7	85.0	-28.3	105.2<br>116.4	36.8	9

[a] Heating and cooling scan was carried out at 10°C/min; [b] Crystallinity was evaluated on the second heating scan and considering ΔHm (100%) = 93.1 J/g [Ref 17 and Ref 18 main text].

Table S5. Absorption maxima and corresponding molar extinction coefficient [1] of CA, FM and DR in $CHCl_3$ and in EtOH 95%

	$CHCl_3$ [2]		EtOH 95% [2]	
Sample	λ_{max} (nm)	ε_{max} (L mol^{-1} cm^{-1})	λ_{max} (nm)	ε_{max} (L mol^{-1} cm-1)
CA	344	4780	343	4230
	330	4426	329	3877
	294	19460	293	17885
	264	21400	261	21145
FM	301	7200	300	6764
	291	6000	289	5086
	268	19300	266	17400
DR	483	30951	507	32408

[1] Molar extinction coefficients were evaluated by calibration curves by collecting spectra at different concentration of the chromophore. [2] Dielectric constants of $CHCl_3$ = 4.81; Dielectric constant of EtOH = 25.30 [Ref 41 main text].

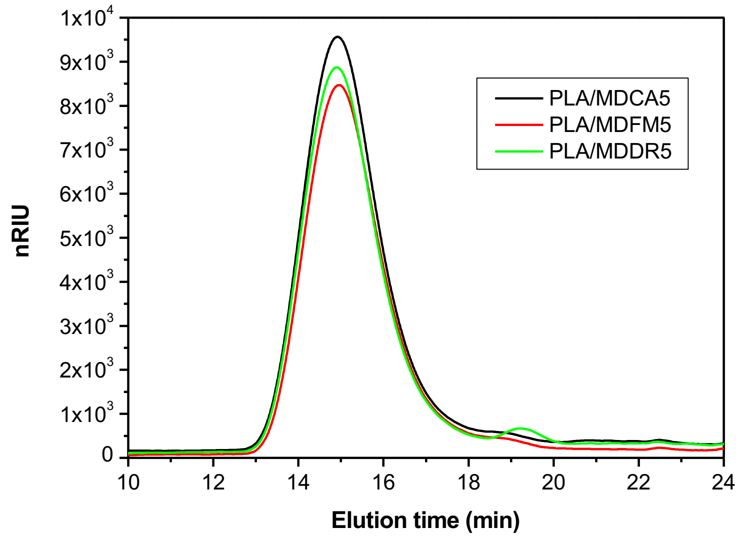

Figure S9. Chromatograms collected during SEC analysis of PLA/MDCA5, PLA/MDFM5 and PLA/MDDR5.

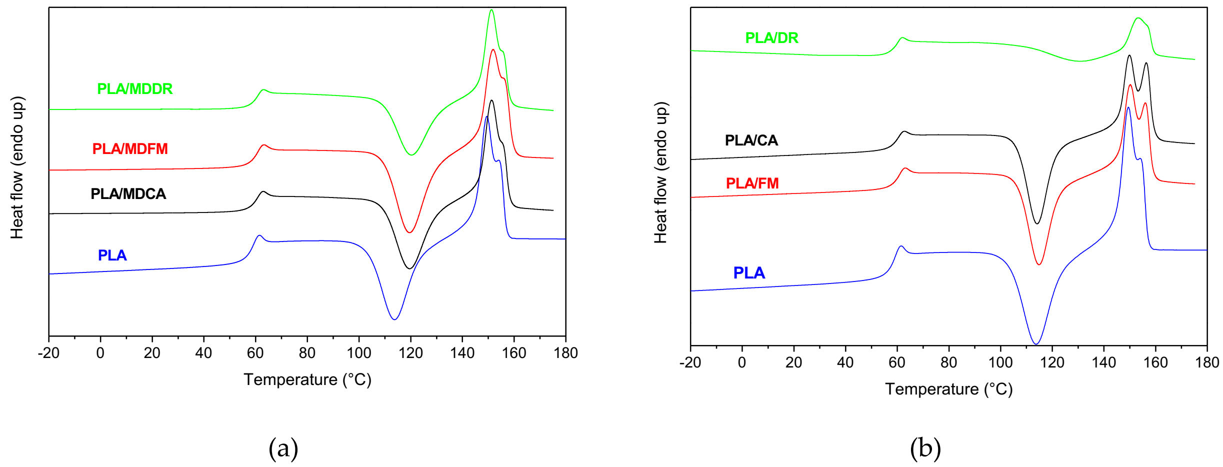

Figure S10. DSC curves (second heating scan): (a) PLA, PLA/MDCA5, PLA/MDFM5 and PLA/MDDR5 and (b) PLA, PLA/CA, PLA/FM and PLA/DR. The curves were vertically shifted for clarity. Heating and cooling scans were carried out at 10°C/min

Table S6. Ratio between the emission intensity of the band at 350 and 360 nm of the spectra of PLA/CA and PLA/MDCA5

Sample	I_{350}/I_{360}
PLA/CA aluminum side	0.86
PLA/CA Teflon side	0.88
PLA/CA aluminum side after annealing	0.72
PLA/CA Teflon side after annealing	0.72
PLA/MDCA5 aluminum side	0.84
PLA/MDCA5 Teflon side	0.85
PLA/MDCA5 aluminum side after annealing	0.72
PLA/MDCA5 Teflon side after annealing	0.72

Microwave assisted Synthesis of Macromolecular Dyes (MD) by using FM as co-initiator

This synthesis was carried out by using FM as co-initiator, reagents in bulk (see the conventional synthesis method in the main text for stoichiometry) are weighted directly into dedicated 10 mL vials and capped with the provided septum without oxygen removal. A CEM Explorer monomodal reactor (max. power output = 300 W) equipped with IR optical thermometer and a 48 positions autosampler was employed in Dynamic Field Tuning mode. Irradiation was auto regulated to achieve the following set temperature gradient: r. T. → 30 °C; 30 °C, isothermal (hold for 1 minute); 30 °C → 60 °C; 60 °C, isothermal (hold for 1 minute); 60 °C → 90 °C; 90 °C, isothermal (hold for 1 minute); 120 °C → 130 °C; 130 °C, isothermal (hold for 20 minutes); 130 °C → r. T. Pressurized air cooling option was disabled in order to enhance homogeneity. Purification was performed as reported for the conventional procedure in the experimental part (main text). Yields ranged from 65% to 85%. The samples are named MDFMmw (where mw is for microwave assisted synthesis) followed by a number corresponding to the percentage of FM with respect to L-lactide. For example, in the case of sample prepared by using 5 mol% of FM with respect to L-lactide and the reaction was microwave assisted the acronym is MDFMmw5.

Table S7. DSC data of MDFMmws [1]

Sample	First heating			Second heating			
	Tm (°C)	ΔH_m (J/g)	Crystallinity (%) [2]	T_{cc} (°C)	ΔH_{cc} (J/g)	T_m (°C)	ΔH_m (J/g)
MDFMmw1	161.1<br>156.1	82.6	88.8	116.0	-45.5	156.0<br>147.0	47.1
MDFMmw3	157.2<br>151.5	60.2	64.7	110.0	-36.4	152.0<br>140.0	41.2
MDFMmw5	146.7<br>140.1	49.2	52.9	113.0	-19.3	145.0<br>137.0	17.0
MDFMmw7	140.5<br>132.6	41.8	44.9	113.0	-7.9	140.0<br>132.0	7.3
MDFMmw10	133.9<br>125.3	30.0	32.2	114.0	-1.2	136.0	1.5

[1] Heating and cooling scans were carried out at 20°C/min. [2] Crystallinity was evaluated on the first heating scan and considering ΔH_m (PLA100%) = 93.1 J/g [Ref 17 and Ref 18 main text].
